# Parvovirus B19 DNA CpG Dinucleotide Methylation and Epigenetic Regulation of Viral Expression

**DOI:** 10.1371/journal.pone.0033316

**Published:** 2012-03-07

**Authors:** Francesca Bonvicini, Elisabetta Manaresi, Francesca Di Furio, Luisa De Falco, Giorgio Gallinella

**Affiliations:** Division of Microbiology, Department of Hematology and Oncological Sciences, University of Bologna, Bologna, Italy; University of Sussex, United Kingdom

## Abstract

CpG DNA methylation is one of the main epigenetic modifications playing a role in the control of gene expression. For DNA viruses whose genome has the ability to integrate in the host genome or to maintain as a latent episome, a correlation has been found between the extent of DNA methylation and viral quiescence. No information is available for Parvovirus B19, a human pathogenic virus, which is capable of both lytic and persistent infections. Within Parvovirus B19 genome, the inverted terminal regions display all the characteristic signatures of a genomic CpG island; therefore we hypothesised a role of CpG dinucleotide methylation in the regulation of viral genome expression.

The analysis of CpG dinucleotide methylation of Parvovirus B19 DNA was carried out by an aptly designed quantitative real-time PCR assay on bisulfite-modified DNA. The effects of CpG methylation on the regulation of viral genome expression were first investigated by transfection of either unmethylated or in vitro methylated viral DNA in a model cell line, showing that methylation of viral DNA was correlated to lower expression levels of the viral genome. Then, in the course of in vitro infections in different cellular environments, it was observed that absence of viral expression and genome replication were both correlated to increasing levels of CpG methylation of viral DNA. Finally, the presence of CpG methylation was documented in viral DNA present in bioptic samples, indicating the occurrence and a possible role of this epigenetic modification in the course of natural infections.

The presence of an epigenetic level of regulation of viral genome expression, possibly correlated to the silencing of the viral genome and contributing to the maintenance of the virus in tissues, can be relevant to the balance and outcome of the different types of infection associated to Parvovirus B19.

## Introduction

Parvovirus B19 (B19V) is a human pathogenic virus, member of the Erythrovirus genus in the Parvoviridae family [1]. Infection is widespread and can be associated with an ample range of pathologies and clinical manifestations, whose characteristics and outcomes depend on the interplay between the virus, the physiological and immune status of the infected individuals and the development of a virus-specific host immune response [2], [3]. The virus shows a selective tropism for erythroid progenitor cells in the bone marrow, exerting a cytotoxic effect and causing a block in erythropoiesis that can manifest as transient or persistent erythroid aplasia [4]. The virus can be transmitted to the fetus, where infection of erythroid progenitors, fetal cardiac myocytes and placental endothelial cells can lead to hydrops fetalis and/or fetal death [5]. Common manifestations of B19V infection are erythema infectiosum in children or post-infection arthropathies mainly in adults, however infection has been implicated in a growing spectrum of other different pathologies affecting diverse tissues and organs, also including a possible involvement in autoimmune diseases [6]. Following primary infection, a characteristic of the virus is its ability to persist in tissues of different origins, mainly bone marrow, liver, heart, synovia and skin, and constituting the so-called bioportfolio [7].

Structural features of B19V are common to viruses in the family [8]. One molecule of linear single-stranded DNA of either positive or negative polarity, about 5600 bases in length, is encapsidated in isometric virions, approximately 25 nm in diameter. The viral genome is composed of a unique internal region, containing all the coding sequences, flanked by two inverted, repeated terminal regions (ITR). Terminal regions are 383 nt. long, the distal 365 forming an imperfect palindrome [9], [10] able to fold in double-stranded hairpin structures that are effective as origins of replication of the viral genome [11], [12]. Expression is driven by the activity of a single promoter proximal to the left end terminal region, then a series of splicing and cleavage/polyadenylation events leads to the production of a full set of viral mRNAs coding, among others, for a viral NS protein and capsid proteins VP1 and VP2 [13], [14]. Functional analysis of the viral genome indicates that transcription and replication are interrelated and that B19V genome can be considered a single, two state functional unit [15], [16]. In such model, onset of transcription directly leads to the production of a full ensemble of viral mRNAs and to replication of the viral genome, which in turn ensures the achievement of a productive replicative cycle.

A productive replicative cycle mainly occurs in erythroid progenitor cells, requiring erythropoietin stimulation [17], while in other cell types replication can be severely restricted [18], [19]. In such cases, possible levels of restriction to viral replication can be the conversion of the parental single-stranded genome into a double-stranded replicative intermediate [18], as well as the effectiveness of the cellular machinery in assisting viral DNA transcription or replication [19]. The unique viral promoter is generic, interacts with cellular factors such as Sp1 and Sp3 and the viral protein NS as transactivator [20], [21], and its activity can be detected in several cell types although is stronger in erythroid progenitors. While it is assumed that viral replication depends on cellular S-phase, it is emerging that expression of the viral genome has itself effects on the expression of cellular factors modulating cell cycle progression [22]. A role in the regulation of the viral replicative cycle can possibly be played by interrelated epigenetic mechanisms active in eukaryotic cells [23], such as DNA methylation, or complexing of the viral genome into nucleosomes, with consequent effects due to nucleosome positioning and histone modifications.

Epigenetic mechanisms, and in particular the impact of cytosine methylation at CpG dinucleotides on the viral life cycle have been mainly studied for viruses that can establish latency and undergo reactivation, such as viruses in the Herpesviridae family, or for viruses that can integrate their genome into the host genome such as Retroviridae or Papillomaviridae. In general, a correlation has been found between the extent of CpG dinucleotides methylation of viral genomes and viral quiescence [24]. Scarce information is on the contrary available on the possible occurrence and role of methylation for actively replicating viruses, and in particular for single-stranded DNA viruses such as viruses of the Parvoviridae family. Parvovirus B19 possesses a complex lifestyle, combining features of both lytic and persistent infections, making it an attractive model for the study of epigenetic levels in the regulation of virus-host interactions.

In the present work, investigation was focused on the occurrence and role of CpG dinucleotides methylation in the DNA of Parvovirus B19 and on a possible epigenetic level of regulation of viral genome expression. In our experimental scheme, the effects of CpG methylation of viral DNA were first investigated by transfection of unmethylated or in vitro methylated viral DNA in a model cell line, then its presence and possible effects assessed in the course of a prolonged in vitro infection in different cellular environments. Finally, the presence of CpG methylation was documented in viral DNA obtained from bioptic samples, indicating the occurrence of this epigenetic modification in the course of natural infections.

## Methods

### Bioinformatic analysis

Parvovirus B19 genome sequence was analysed for the presence and distribution of CpG dinucleotides by means of EMBOSS CpGPlot, a web-based program available at EMBL-EBI [25]. Submission consisted of a consensus sequence of B19 genome, genotype 1, using standard parameters (Window length 100, Obs/Exp CpG ratio 0.6, Min C+G 50%, Min Length 200).

### Plasmid DNA and in vitro DNA methylation

The plasmid clone pB19-M20, generated as a complete genomic clone from J35 virus source (GenBank ID: AY386330), was previously described [26] and is a kind gift of Kevin Brown. The full-length clone pB19-M20 was digested with SalI-HF (New England Biolabs) to release the B19V genome insert from the vector and the DNA fragments were separated by agarose gel electrophoresis. The 5.6 Kb B19V genome was excised and purified by using the Wizard SV Gel and PCR Clean-Up System (Promega). The plasmid clone pEGFP-P6 has been constructed by PCR amplification of the segment between nt. 233 and 582, using RE modified primers, and cloning in pEGFP-1 (Invitrogen) as an XhoI-BamHI insert. pEGFP-P6 plasmid DNA was then linearized by XhoI, dephosphorylated by alkaline phosphatase treatment, and cleaved by BamHI to release the B19V insert, containing the viral P6 promoter, from the vector backbone, containing the EGFP reporter gene (all enzymes from New England Biolabs). DNA fragments were separated by agarose gel electrophoresis, excised and purified as described. Following in vitro methylation of either B19V insert or vector backbone (as described below), methylated or unmethylated DNA segments were ligated in different combinations (T4 DNA Ligase, New England Biolabs). Linear products consisting of the viral promoter religated upstream of the reporter gene were separated by agarose gel electrophoresis, excised and purified as described.

Excised B19V DNA, pEGFP-P6 plasmid DNA and viral promoter insert and vector backbone from pEGFP-P6 underwent in vitro methylation with CpG methyltransferase (M.SssI; New England Biolabs). An amount of 1 µg of target DNA was twice treated with 4 units of M.SssI enzyme for 2 hours at 37°C in a 20 µl reaction volume containing 1.6 mM of S-adenosylmethionine and NEBuffer 2. Enzyme inactivation was carried out by treatment at 65°C for 20 min. Methylase treated DNA was purified by using the Wizard SV Gel and PCR Clean-Up System (Promega). DNA was modified by bisulfite treatment, using the MethylCode™ Bisulfite Conversion Kit (Invitrogen) following the manufacturer's instructions. Successful methylation (min. 99% methylation of CpG dinucleotides) and complete conversion following bisulfite treatment was confirmed by using methylation-specific Real Time PCR assay and sequencing (see below).

### Bisulfite Specific and Methylation Specific Real Time PCR

PCR primers were specifically designed to amplify unmethylated or methylated bisulfite-modified B19 DNA, but not unmodified DNA. BSP (bisulfite specific PCR) primers amplify bisulfite-converted sequences independently of the methylation status, MSP (methylation specific PCR) primers selectively amplify bisulfite-converted sequences depending on the previous CpG methylation status (Table 1).

**Table 1:** Primers for bisulfite specific and methylation specific PCR.

Primer	Sequence (5′-3′)	Sense	Target
0218	G**T**TGG**TTT**AGAG**TT**AA**TTT**TAATT**T**	forward	common to bisulfite-modified DNA
0350 UnMet	**A**CC**AA**TC**A**CC**A**CC**AA**TA**AA**C**A**	reverse	specific for unmethylated bisulfite-modified DNA
0350 Met	GCCG**A**TCGCCGCCG**A**TA**AA**CG	reverse	specific for methylated bisulfite-modified DNA
0423	AAAT**AA**CT**A**CCCATTT**A**CATAA**AA**C	reverse	common to bisulfite-modified DNA

In **bold** are shown bases selectively matching to bisulfite-modified DNA:

C→**T** on sense target strand.

G→**A** on complementary strand.

Primers for bisulfite specific and methylation specific PCR are designed on bisulfite-converted, positive-sense strand of B19V genome and can be common and independent of CpG methylation status of target (0218, 0423), or be specific for unmethylated or methylated targets (0350 UnMet, 0350 Met).

For real-time PCR, bisulfite-modified or native DNA was amplified by using QuantiTect SYBR Green PCR Kit (Qiagen) containing a final concentration of 0.5 µM of each primer in a final volume of 20 µl. The PCR was performed using the RotorGene 3000 system (Corbett Research) with a 95°C activation step for 15 min; 95°C for 30 sec, 57°C for 30 sec and 72°C for 15 sec, coupled to signal acquisition, for 40 cycles. Melt of the PCR product was performed from 60 to 90°C, rising in 0.5°C increments, waiting for 30 sec at the first step and for 5 sec at each step thereafter, and acquiring the fluorescence at each temperature increment. Fluorescence emission was recorded in the FAM/Sybr channel of the instrument and analyzed by using the quantitation functions available in the RotorGene 6.0 software. Melting analysis was used for the determination of the specificity of the amplification products by defining, for each reaction, the melting profile and the Tm of the products; specific accumulation of the amplification products was also confirmed by agarose gel electrophoretic analysis.

### Transfection and Infection Experiments

#### Cells

UT7/EpoS1 cells, kindly obtained form K.Sugamura [27], were cultured in IMDM (Cambrex), supplemented with 10% FCS and 2 U/ml Epo, at 37°C and 5% CO_2_. U937 cells [28], obtained from LGC Standards (Italy), were cultured in RPMI (Cambrex) supplemented with 10% FCS. Cells were kept in culture at densities between 2×10^5^–1×10^6^ cells/ml, and used for both transfection and infection experiments when at a density of 3×10^5^ cells/ml.

#### Transfection

Transfection of UT7/EpoS1 cells was performed by using the Amaxa Nucleofector II system (Lonza), using Nucleofector Reagent R and setting the program T20. An amount of 1×10^6^ cells was transfected with 1 µg of test DNA. Following transfection, the cells were incubated at 37°C in complete medium at an initial density of 10^6^ cells/ml. Constant volumes of cell cultures, corresponding to 0.5 ml of the initial cell culture volume, were collected at different time points from 2 to 72 hours post-transfection (hpt) for nucleic acid purification and analysis.

#### Infection

For infection, both UT7/EpoS1 and U937 cells were incubated at a density of 10^7^ cells/ml in minimal medium in the presence of a reference viremic serum, in order to obtain a multiplicity of infection of 10^3^ geq/cell. Following adsorption for 2 h at 37°C, the inoculum virus was washed and the cells were incubated at 37°C in complete medium at an initial density of 10^6^ cells/ml. Constant volumes of cell cultures, corresponding to 0.5 ml of the initial cell culture volume, were collected at different time points from 0 to 3 days post-infection (dpi). Thereafter, equivalent volumes of cell cultures were harvested at three days intervals post-infection (6–48 dpi), every time before adding three time volumes of fresh culture medium to support cellular growth.

#### Nucleic acid purification

Cell culture samples, collected at the different time points following transfection or infection, were divided in aliquots each corresponding to 1×10^5^ cells for transfection and 5×10^5^ for infection experiments, then centrifuged at 12000 g for 2 min. Pelleted cells were processed both by using the QIAamp DNA Mini Kit (Qiagen), in order to obtain a total nucleic acid fraction, and by using the ToTally RNA purification kit (Ambion), in order to obtain a nucleic acid fraction enriched in RNA. For methylation analysis, purified DNA was bisulfite modified by using the MethylCode™ Bisulfite Conversion Kit (Invitrogen) following the manufacturer's instructions.

### Quantitative PCR analysis

#### Standards and Primers

Standard targets for the amplification reactions were obtained from plasmid pHR0 [29], that contains an insert corresponding to the complete internal region of B19 virus genome (nt. 345–5245). Primers for the amplification of viral targets were the pair R2210–R2355, able to amplify viral DNA and total viral RNA [15], [16]. For normalisation with respect to the number of cells, a target sequence in the region of genomic 18S rDNA was selected and amplified by using the primers 18Sfor (CGGACAGGATTGACAGATTG) and 18Srev (TGCCAGAGTCTCGTTCGTTA). Oligonucleotides were obtained from MWG Biotech.

#### Quantitative real-time PCR

For the quantitative analysis of viral DNA, an aliquot of the total nucleic acid fraction, corresponding to 0.1 volumes of experimental samples, was directly amplified, while for the analysis of methylation status of viral DNA, an aliquot corresponding to 0.2 volumes of experimental samples was first processed by bisulfite treatment, then amplified in parallel amplification reactions for determination of methylated or unmethylated fractions. For the quantitative analysis of viral RNA, an aliquot of the RNA enriched fraction corresponding to 0.1 volumes of experimental samples was first treated with Turbo DNAfree reagent (Ambion) and then amplified. Quantitative real-time PCR and RT-PCR were carried out by using the RotorGene 3000 system (Corbett Research) and SybrGreen detection of amplification products. Amplification reactions were performed by using QuantiTect PCR SybrGreen PCR Kit (Qiagen) or QuantiTect SybrGreen RT-PCR Kit (Qiagen), including 10 pmol of each specific primer pair. For PCR, thermal profile consisted in 15 min at 95°C, then 40 cycles of 15 sec at 95°C, 30 sec at 55°C, and 30 sec at 72°C coupled to signal acquisition. For RT-PCR, two parallel reactions were performed for each sample, either including (RT+) or omitting (RT−) the reverse transcriptase from the reaction mix, and performing an initial step consisting in 30 min at 50°C, before the amplification reaction with a standard thermal profile. A final melting curve was performed, with thermal profile ramping from 60°C to 90°C rising in 0.5°C increments, waiting for 30 sec at the first step and for 5 sec at each step thereafter, and acquiring the fluorescence at each temperature increment. Quantitation of total viral DNA, of methylated/unmethylated fractions of viral DNA, and of total viral RNA was obtained by the absolute quantitation algorithm, using calibration curves obtained from respective standard targets and normalized by the amount of rDNA.

### Biological specimens

Two groups of bioptic samples were analysed. One group included 14 samples, obtained from routine diagnostic analysis, previously proved positive for the presence of B19V DNA [29], with productive infection documented by in situ hybridisation for the detection of viral nucleic acids and indirect immunofluorescence for the detection of viral capsid proteins [18]. Another group included 11 skin biopsies harbouring B19V DNA, without any evidence of productive viral infection.

Bioptic samples were processed for DNA extraction by using the QIAamp DNA Mini Kit (Qiagen). For the determination of the amount of viral DNA, an aliquot of the total nucleic acid fraction, corresponding to 0.1 volumes of experimental samples, was directly amplified as described. For the analysis of the methylation status of viral DNA, a maximal amount of 500 ng of purified DNA underwent bisulfite treatment, then an aliquot corresponding to 0.2 volumes of initial samples was amplified using BSP or MSP primers as described.

### Ethics statement

Clinical samples and data used in this study were available to our research group as part of the institutional diagnostic service at the Microbiology Unit, Policlinico S.Orsola-Malpighi, Bologna. All analysed samples have been sent from the Surgical Pathology Department of the Policlinico S.Orsola-Malpighi to our laboratory with a specific request of detection of B19V DNA, and have been collected, processed, assigned an anonymous code number and archived according to approved and certified diagnostic protocols. Investigation described in this study was carried out on archived residual specimens stored for research use following diagnostic analysis and, in accordance with the recommendations of the Ethical Committee of Policlinico S.Orsola-Malpighi, did not require collection of informed consent. All analysed samples are anonymous and unlinked from clinical databases, in accordance with Italian Privacy Law (Decree 196/2003, §110).

## Results

### Parvovirus B19 genome and CpG islands

**Figure 1:**
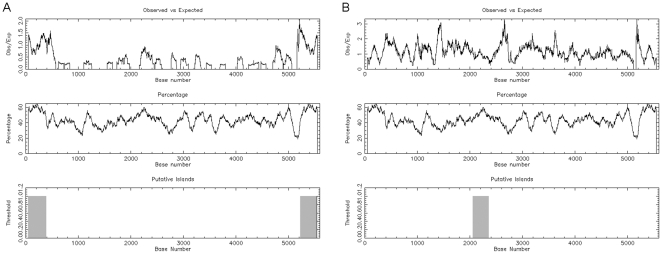
Identification of CpG islands within B19V genome. Analysis of Parvovirus B19 genome sequence for the presence and distribution of CpG dinucleotides was performed by means of EMBOSS CpGPlot. Submission consisted of a consensus sequence of B19 genome, genotype 1, using standard parameters (Window length 100, Obs/Exp CpG ratio 0.6, Min C+G 50%, Min Length 200). Results are shown for positive sense (A) and complementary (B) strands. Upper graphs, distribution of observed/expected ratios of CpG dinucleotides; middle graphs, distribution of C+G; lower graphs, identification of putative CpG islands according to set parameters. (EMBOSS CpGPlot accessed at http://www.ebi.ac.uk/Tools/emboss/cpgplot/).

As a basis for investigation, Parvovirus B19 genome sequence was inspected for the presence and distribution of CpG dinucleotides by means of EMBOSS CpGPlot, a web-based program available at EMBL-EBI. Submission of a consensus sequence of B19V genome, genotype 1, for analysis by CpGPlot using standard parameters indicated that the inverted terminal regions (nt. 1–383 and nt. 5214–5596) on the positive sense strand and the central exon region (nt. 2060–2355) on the complementary strand present a clustered CpG dinucleotide distribution, a pattern typical of CpG islands [25] (Figure 1).

**Figure 2:**
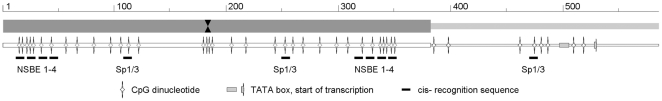
Presence and distribution of CpG dinucleotides in B19V genome. The left terminal region and the proximal part of the internal region of B19V genome are shown in the diagram (nt.1–586, scale at the top line). Upper line: thick segment, left terminal region and axis of dyad symmetry; thin segment, internal region. Middle line: distribution of CpG dinucleotides, TATA box and start of transcription. Lower line: distribution of cis- recognition sequence elements. NSBE 1–4, NS protein binding elements 1–4 [19]. Sp1/3, Sp factors 1/3 recognition sequences.

Sequence inspection within the left terminal region highlighted the distribution of CpG dinucleotides (Figure 2). Within a 383 bps sequence of dyad symmetry, a set of 34 CpG dinucleotide pairs are present, comprising a cluster at the site of hairpin symmetry (nt. 179–188) and two symmetric clusters at distal positions (nt. 14–28 and 338–352), in addition to other disperse elements within hairpin. The distal symmetric clusters are close to cis- recognition sequences, such as Sp1 and Sp3 factors binding sites (classical GC boxes) [20] and viral NS protein binding elements [21]. Additional CpG dinucleotides are present outside the left terminal region as part of the viral promoter, within the transcription start site at nt. 531 (8/148 bps). On the contrary, only sparse CpG dinucleotides are present in the central exon region (9/274 bps). We therefore focused our attention on the occurrence of cytosine methylation in CpG dinucleotides within the left terminal region and its possible role on regulation of gene expression, investigating the region between the internal harm of the palindrome and the end of ITR (nt. 183–383).

### Methylation-specific quantitative PCR assay

Analysis of CpG DNA methylation within the selected target region was carried out by an aptly designed methylation-specific, quantitative real-time PCR assay on bisulfite-modified DNA.

For validation of the assay, a B19V DNA standard target was obtained as an excised insert from pB19-M20 plasmid [26] and in vitro methylated at CpG dinucleotides by means of M.SssI enzyme. Both native and in vitro methylated DNA were then subjected to bisulfite treatment, which converts unmethylated C bases into T, thus originating two noncomplementary strands. All primers for quantitative PCR, described in Table 1, were subsequently designed for amplification of the sense strand, and employed as BSP (bisulfite specific PCR) primer pairs, able to specifically amplify bisulfite-converted sequences independently of the methylation status, and as MSP (methylation specific PCR) primer pairs, used in two different combinations able to selectively amplify bisulfite-converted sequences depending on the previous CpG methylation status of complementary bases. The analytical performance of the BSP and MSP assays was evaluated on serial dilutions of native and M.SssI methylated pB19-M20 targets, either untreated or bisulfite-converted, and is reported in Table 2. BSP assay specifically amplified bisulfite-converted DNA with respect to untreated, with similar efficiencies independently of the methylation status, while MSP assay showed selective amplification of unmethylated or methylated targets depending on the primer combinations used. For MSP assay, a quantitative evaluation of methylated [DNA_met_] or unmethylated [DNA_unmet_] targets was obtained by direct interpolation on respective calibration curves. A methylation index was defined as the ratio of methylated over total DNA [DNA_met_/(DNA_met_+DNA_unmet_)], as a measure of the methylation status on B19V DNA. BSP and MSP amplification products also showed different melting profiles indicative of a different base content resulting from preservation of methylated cytosines following bisulfite treatment, therefore allowing confirmation of selective amplification of methylated or unmethylated viral DNA. Finally, BSP amplification products were sequenced, and the resulting chromatograms both indicated the complete conversion operated by bisulfite treatment on unmethylated C bases, and identified the unconverted methylated C bases in CpG dinucleotides.

**Table 2:** Analytical characteristics of bisulfite and methylation specific real-time, quantitative PCR.

Target^a^	Primer Pair	Detection range	Efficiency	Tm
Unmethylated	BSP 0218-0423	10^2^–10^8^	1.85	74.80
	MSP 0218-0350 UnMet	10^2^–10^8^	1.84	73.15
	MSP 0218-0350 Met	>10^8^	–	–
Methylated	BSP 0218-0423	10^2^–10^8^	1.90	77.00
	MSP 0218-0350 UnMet	>10^4^ b	–	74.00^b^
	MSP 0218-0350 Met	10^2^–10^8^	1.83	76.80

a) bisulfite-treated B19V insert excised from pB19-M20.

b) amplification of residual, unmethylated target following in vitro M.SssI treatment.

Unmethylated or in vitro methylated, bisulfite-treated B19V inserts from pB19-M20 plasmid have been quantified spectrophotometrically and 10-fold diluted to obtain quantitative standards for the definition of calibration curves. Analytical characteristics have been determined from triplicate amplification of standards in the range 10^2^–10^8^ target copies/reaction. For calibration curves, R^2^ is >0.99 and CV is 0.16–0.24.

### CpG DNA methylation and viral genome expression

The effects of the occurrence of CpG methylation of viral DNA on the viral expression profile was investigated by transfection of unmethylated or in vitro methylated viral DNA in the UT7/EpoS1 model cell line, able to support both transcriptional and replicative activity of a transfected B19V complete genomic insert [12], [26], and subsequent quantitative analysis of viral nucleic acids in a time course post-transfection. For this purpose, UT7/EpoS1 cells were transfected with 1 µg/10^6^ cells of either unmethylated or M.SssI methylated B19V DNA, previously excised from pB19-M20 plasmid, using the Amaxa nucleofection system. Constant volumes of cell cultures, corresponding to 0.5 ml of the initial cell culture volume, were collected from 2 to 72 hours post-transfection (hpt). For quantitative analysis of viral nucleic acids, total DNA and RNA from cell fractions were purified and viral DNA or RNA were amplified by using previously established primer pairs and PCR conditions. Purified DNA was bisulfite-modified, then viral bisulfite-modified DNA was amplified by using the MSP primer pair for analysis of the methylation status of viral DNA. Data were analysed by using the quantitation analysis and melting curve analysis algorithms in the RotorGene 6.0 software, calibrated to standard reference curves.

Results obtained from a quantitative analysis of viral nucleic acids are reported in Table 3. Transfected unmethylated viral DNA was not methylated to significant fractions, while transfected methylated viral DNA was demethylated to a marginal extent, with a calculated methylation index varying in the range 0.97–0.93. Transcriptional activity of transfected viral DNA indicated a possible correlation with the methylation status, as methylated compared to unmethylated viral DNA showed a 1–1.5 Log reduction in the ratio of total viral RNA to viral DNA.

**Table 3:** DNA methylation and quantitative analysis of B19V nucleic acids in transfection experiments.

Transfection	Sample	B19 DNA	Met Index	B19 RNA	RNA/DNA
pB19-M20, Unmethylated	02 hpt	1.44E+06	<0.001	8.32E+05	5.78E−01
	24 hpt	1.49E+06	<0.001	3.86E+07	2.60E+01
	48 hpt	5.49E+05	<0.001	1.17E+10	2.13E+04
	72 hpt	1.23E+05	<0.001	1.34E+10	1.09E+05
pB19-M20, Methylated	02 hpt	1.14E+06	0.969	2.42E+04	2.12E−02
	24 hpt	1.09E+06	0.972	1.99E+06	1.82E+00
	48 hpt	1.03E+06	0.955	2.19E+09	2.13E+03
	72 hpt	2.56E+05	0.929	1.58E+09	6.18E+03

UT7/EpoS1 cells were transfected with 1 µg/10^6^ cells of either unmethylated or M.SssI methylated B19V DNA, excised from pB19-M20 plasmid, and samples collected at 2, 24, 48, 72 hours post-transfection (hpt). Quantitative analysis was carried out for the determination of the amounts of viral DNA and total viral RNA from cell fractions (copies/10^4^ cells). Methylation index was calculated by parallel amplification with both MSP primer pairs on bisulfite-modified DNA and is expressed as ratio of methylated over total viral DNA.

**Figure 3:**
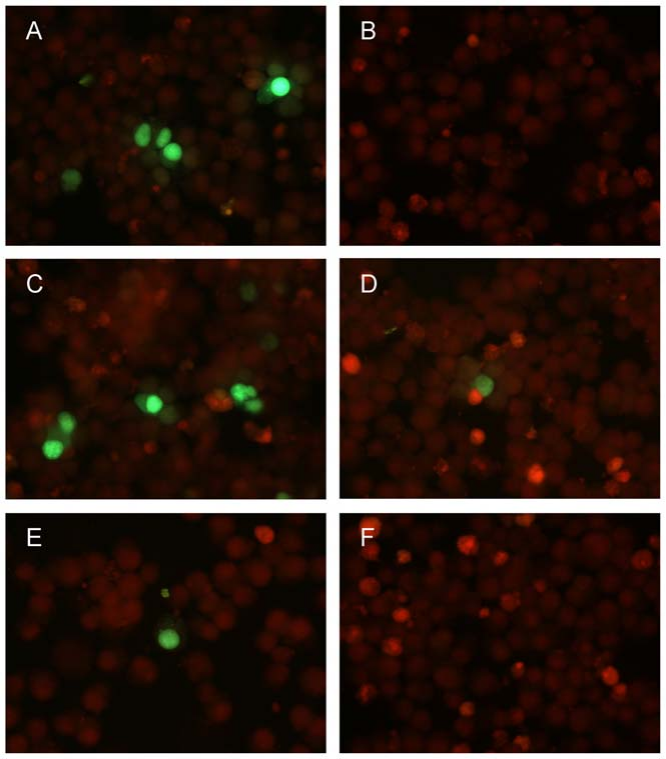
CpG methylation and EGFP reporter gene expression in UT7/EpoS1 cells. Activity of viral P6 promoter, as determined by direct detection of EGFP fluorescence following transfection in UT7/EpoS1 cells of: unmethylated (A) or in vitro methylated (B) pEGFP-P6 plasmid DNA; linear products derived from ligation of the viral promoter (unmethylated, C and E, or methylated, D and F), upstream of the reporter gene in pEGFP-1 vector backbone (unmethylated, C and D, or methylated, E and F). Transfected UT7/EpoS1 cells were harvested at 72 hpt, about 50000 cells were spotted on glass slides, fixed with 4% paraformaldehyde in PBS, and counterstained with Evans Blue. FITC filter set, original magnification 40×.

A possible role of CpG methylation within the left terminal region of B19 genome was directly investigated by analysing the activity of the viral promoter in directing the expression of a reporter gene. For this purpose, an insert corresponding to the nt. 183–530 of viral DNA and encompassing the viral P6 promoter was cloned in pEGFP-1 plasmid vector to give the plasmid pEGFP-P6. Purified plasmid DNA was methylated in vitro at CpG dinucleotides by means of M.SssI enzyme, then both unmethylated and in vitro methylated DNA were transfected in UT7/EpoS1 cells as described before, with equal efficiency as determined by quantitative PCR. The activity of the reporter gene was determined by microscopic evaluation of EGFP fluorescence. EGFP expression was observable for unmethylated but not for methylated pEGFP-P6, suggesting a possible direct effect of CpG methylation on hampering viral promoter activity (Figure 3, A–B).

To assess a specific effect of methylation of CpG dinucleotides within the viral promoter with respect to intragenic CpG dinucleotides in the EGFP sequence, the B19V insert containing the viral P6 promoter was released from the vector backbone containing the EGFP reporter gene. Then, DNA fragments were separately methylated in vitro at CpG dinucleotides by means of M.SssI enzyme and different combinations of methylated or unmethylated B19V insert or vector backbone segments were ligated, yielding linear products consisting of the viral promoter religated upstream of the reporter gene. Purified ligation products were transfected in UT7/EpoS1 cells and the activity of the reporter gene determined as before. EGFP expression was observable to levels comparable to unmethylated plasmid in the case of ligated unmethylated viral promoter and vector backbone, was reduced to minimal levels following methylation of either the viral promoter or the vector backbone, and was absent when both of them were methylated (Figure 3, C–F). These results indicate, in addition to a non-specific effect of intragenic CpG methylation on gene expression, that methylation of CpG dinucleotides within the viral promoter can have a direct negative effect on its activity.

### CpG DNA methylation in a time course of in vitro infection

The effects of the presence of methylated CpG dinucleotides in viral DNA in modulating genome expression profile prompted for investigation of a possible role of DNA methylation in the course of infection of model target cells. For this purpose, the occurrence of CpG DNA methylation in B19V DNA and the possible effects on the viral expression profile were investigated in a prolonged course of in vitro infection in two different cell lines, namely, UT7/EpoS1 cells, chosen as a restrictive model cell system [15], [16], and U937 cells, proposed as a nonpermissive model cell system [30].

UT7/EpoS1 and U937 cells were infected with a reference viremic serum at a calculated multiplicity of infection of 10^3^ geq/cell and maintained at an initial density of 10^6^ cells/ml. Constant volumes of cell cultures were collected at daily intervals from 0 to 3 days post-infection, then equivalent volumes of cell cultures were harvested at three days intervals post-infection until day 48 post-infection, every time before adding three time volumes of fresh culture medium to support cellular growth. Quantitative analysis of viral (unmethylated/methylated) DNA and RNA was then carried out as described for the transfection experiments.

Results obtained from a quantitative analysis of viral nucleic acids are reported in Table 4. Input viral DNA was characterized by a minimal but measurable amount of methylated CpG dinucleotides, with a calculated methylated index at about 10^−4^. In the course of infection, the differences in the viral replicative cycles were evident and a progressive, differential methylation of intracellular viral DNA was observed throughout the time course of infection in the two model cell systems.

**Table 4:** DNA methylation and quantitative analysis of B19V nucleic acids in a time course of infection.

Infection	Sample	B19 DNA	Met Index	B19 RNA	RNA/DNA
UT7/EpoS1 cells	00 dpi	8.63E+04	<0.001	1.81E+03	2.10E−02
	01 dpi	1.46E+05	<0.001	2.23E+08	1.53E+03
	03 dpi	1.18E+08	<0.001	3.46E+05	2.93E−03
	06 dpi	7.19E+08	0.002	4.04E+06	5.62E−03
	09 dpi	2.22E+08	0.001	9.80E+06	4.41E−02
	12 dpi	1.99E+08	0.001	7.95E+05	3.99E−03
	15 dpi	5.56E+06	0.001	1.33E+05	2.39E−02
	20 dpi	4.21E+05	0.002	1.85E+04	4.39E−02
	24 dpi	1.12E+05	0.003	8.46E+03	7.58E−02
	27 dpi	1.39E+04	0.031	1.29E+02	9.28E−03
U937 cells	00 dpi	8.98E+05	0.009	3.79E+03	4.22E−03
	01 dpi	3.93E+04	0.009	3.68E+03	9.36E−02
	03 dpi	4.77E+04	0.008	ND	ND
	06 dpi	2.20E+04	0.013	ND	ND
	09 dpi	3.85E+05	0.041	1.70E+03	4.42E−03
	12 dpi	2.16E+05	0.168	ND	ND
	15 dpi	2.61E+04	0.205	2.49E+03	9.54E−02
	20 dpi	1.01E+04	0.229	ND	ND
	24 dpi	3.63E+03	0.336	1.51E+03	4.16E−01
	27 dpi	2.37E+03	0.423	ND	ND

UT7/EpoS1 and U937 cells were infected with B19V at a multiplicity of infection of 10^3^ geq/cell, and samples collected from 0 to 27 days post infection (dpi). Quantitative analysis was carried out for the determination of the amounts of viral DNA and total viral RNA from cell fractions (copies/10^4^ cells). Methylation index was calculated by parallel amplification with both MSP primer pairs on bisulfite-modified DNA and is expressed as ratio of methylated over total viral DNA. ND: not detected.

In UT7/EpoS1 cells, viral DNA was consistently detected until day 27, with higher levels indicating viral DNA replication occurring through days 2–15. Methylation analysis indicated the presence of low levels of methylated CpG dinucleotides along with a progressive increase in the fraction of methylated DNA, with a calculated methylation index <0.01 for the early time points increasing to a maximal value of 0.03 at day 27. Viral RNA was reliably detected until day 24, showing higher levels indicative of a maximal transcriptional activity occurring through days 1–9.

In U937 cells, viral DNA was also detected until day 27, however with constantly decreasing amounts indicating absence of DNA replication. Methylation analysis indicated the presence of higher levels of methylated CpG dinucleotides compared to UT7/EpoS1 cells, also in this case with a progressive increase in the fraction of methylated DNA, leading from a calculated methylation index of about 0.01 for the early time points, increasing from day 12 onward and reaching a maximal value of 0.34–0.42 at days 24–27. In U937 cells, viral RNA was not reliably detected over background levels throughout the time course, indicating absence of effective transcriptional activity in this nonpermissive cellular system.

### Occurrence of CpG DNA methylation in clinical samples

The presence of CpG DNA methylation in B19V DNA was investigated in two groups of bioptic samples. One group included 14 samples, obtained from routine diagnostic analysis, previously proved positive for the presence of B19V DNA, with productive infection documented by in situ hybridisation for the detection of viral nucleic acids and indirect immunofluorescence for the detection of viral capsid proteins. Another group included 11 skin biopsies harbouring B19V DNA, without any evidence of productive viral infection.

**Figure 4:**
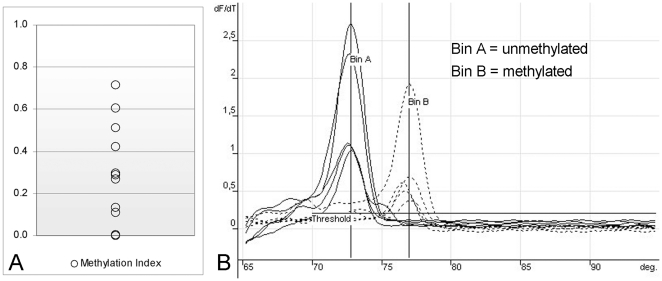
Detection of CpG methylation in B19V DNA from bioptic samples. A. Distribution of methylation indexes of viral DNA present in bioptic samples, as calculated by means of MSP assay [DNA_met_/(DNA_met_+DNA_unmet_]. B. Melting profiles of the amplification products obtained by either MSP primer pair, specific for methylated or unmethylated target sequences (shown for standards and samples with methylation index >0.40).

**Figure 5:**
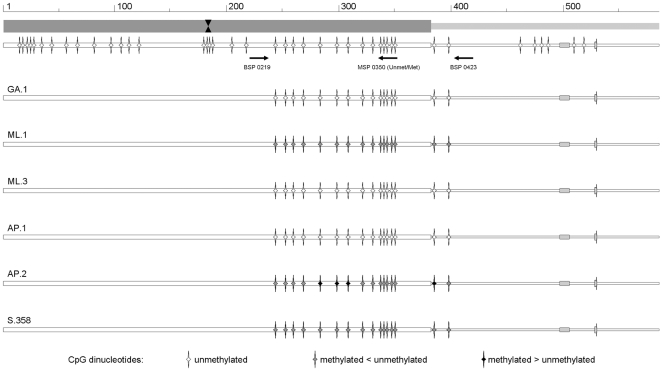
Distribution of CpG methylation in B19V DNA from bioptic samples. Representative diagrams derived from sequencing chromatograms of the amplification products obtained by means of BSP primers on viral DNA from bioptic samples. Top line, schematic diagram of the left terminal region and proximal part of the internal region of B19V genome, as in Figure 2. BSP and MSP primer positions are indicated. Bottom lines, diagrams indicating the methylation status of CpG dinucleotides as determined by sequencing of BSP amplification products, for the selected samples. The relative abundance of methylated or unmethylated cytosines within CpG dinucleotides is indicated by the gray scale code shown at the bottom of the figure.

Within the group of samples obtained from patients with documented infection (Table 5), 12 samples could be amplified by using both BSP and MSP primers following bisulfite treatment of purified DNA. Amplification with both combinations of the MSP primers indicated the presence of detectable levels of CpG methylation in a subset of samples. The distribution of the calculated methylation index for the different samples is reported in Figure 4 and the distribution of methylated CpG dinucleotides within the region of interest is reported in schematic diagram form in Figure 5.

**Table 5:** CpG DNA methylation in B19V DNA present in clinical samples.

Sample (Date)	Tissue	Clinical setting	Viral load	Met Index
A.29 (07/09/2008)	Placenta	IUFD	1.36E+03	0.111
GA.1 (06/07/2006)	Placenta	IUFD	6.33E+06	<0.001
BE.1 (12/06/2007)	Duodenum	Aplastic crisis	6.97E+03	0.005
AP.1 (02/03/2005)	Ileum	IBD	ND	ND
AP.2 (20/10/2005)	Ileum	IBD	ND	ND
CM.1 (15/01/2009)	Myocardium	Myocarditis	1.08E+03	0.136
CM.2 (18/02/2009)	Myocardium	Myocarditis	3.17E+02	0.287
FV.1 (19/04/2010)	Myocardium	Myocarditis	7.62E+03	0.511
ML.1 (21/02/2005)	Myocardium	Myocarditis	2.43E+02	0.268
ML.2 (23/02/2005)	Myocardium	Myocarditis	1.06E+03	0.296
ML.3 (09/03/2005)	Myocardium	Myocarditis	6.09E+04	0.004
MN.1 (08/11/2005)	Myocardium	Myocarditis	4.00E+03	0.423
MN.2 (23/11/2005)	Myocardium	Myocarditis	2.33E+03	0.714
MN.3 (04/01/2007)	Myocardium	Myocarditis	4.76E+03	0.605

Clinical samples, amount of viral DNA present in tissues (genome copies/100 ng cellular DNA) and analysis of methylation status of viral DNA as determined by MSP assay. IUFD: intrauterine fetal death; IBD: inflammatory bowel disease; ND: not determined.

In particular, higher levels of CpG methylation were detected in endomyocardial tissues obtained from patients with myocarditis. When available, follow-up samples in these cases indicated both the persistence of viral DNA and the presence of CpG methylation at stable levels, in the range 0.14–0.71. As an exception, samples ML.1 and ML.2, obtained at presentation, characterized by a similar methylation index of 0.27–0.30, can be compared with sample ML.3 obtained at +18 days, showing an increasing amount of viral DNA and undetectable levels of CpG methylation. Such difference could also be confirmed by sequencing of BSP amplification products, that showed the presence of partially protected C bases adjacent to G, as indicative of the occurrence of CpG methylation, in sample ML.1 but not ML.3. Lower or undetectable CpG methylation levels were observed in two placental samples [31] and in a duodenal tissue sample obtained from a case of acute aplastic crisis [32]. This last sample can be compared to a pair of intestinal samples from a case of chronic inflammatory bowel disease with involvement of B19 virus [33], obtained in the early, active (sample AP.1) and remission (sample AP.2) phases of disease. For these samples, due to limited amounts available, we could perform BSP amplification only, however sequencing of amplification products indicated the occurrence of CpG DNA methylation in B19V DNA present in tissues obtained in the later rather than the early phase of infection. Finally, due to the low amounts of viral DNA, the group of 11 skin bioptic samples harbouring viral DNA [34] was analysed by using BSP primers only. Amplification products could be obtained for 4 samples, while readable sequencing was possible for one sample (S.358, Figure 5), confirming in this case the presence of a fraction of methylated CpG dinucleotides.

## Discussion

DNA methylation at cytosine bases in the context of CpG dinucleotides is one of the main epigenetic modifications, along with histone modifications and nucleosome positioning, playing a role in the control of gene expression. Epigenetic modifications have been extensively studied as markers of neoplastic processes, neurological disorders and autoimmune diseases [23]. The role of epigenetic modifications, in particular CpG methylation, in the genome of DNA viruses has been the subject of studies concerning large DNA viruses, whose genome has the ability to integrate in the host genome or to maintain itself as a latent episome, and can directly or indirectly be associated with the establishment of tumorigenic processes [35]. On the other hand, the occurrence and a possible role of CpG methylation in the genome of small viruses have not been investigated in detail. In particular, little information is available on epigenetic modifications of parvoviral genomes such as AAVs or their derived transduction vectors, and no information is available regarding the human pathogenic parvovirus B19 [24].

Among viral genomes, alike the human genome, the frequency of CpG dinucleotides, specific targets for methylation of cytosine bases, is lower than expected [24]. In the human genome CpG dinucleotides cluster in regions, “CpG islands”, quite rare (1% of the human genome) but critically linked to regulation of the gene expression. Within human parvovirus B19 genome, the inverted terminal regions constitute about 13% of the genome and are essential as origin of replication [12], and, for the left ITR, as part of the unique promoter [36]. The ITRs display all the characteristic signatures of a genomic CpG island [25], and it was possible to hypothesise a role of CpG dinucleotide methylation in the regulation of viral genome expression. In fact, as CpG methylation is involved in gene silencing and silencing of foreign DNA elements integrating in a host genome [37], CpG methylation of parvovirus B19 DNA might also be relevant as a mechanism contributing to silencing of a potentially lytic virus.

Parvovirus B19 has a selective tropism for erythroid progenitor cells, that can be infected giving rise to a productive infection [38]. In these instances, the virus can replicate in a high proportion of cells, causing disregulation of cell cycle progression, block of erythroid differentiation and apoptosis of infected cells [22]. Alternatively, the virus can persist in a variety of tissues, such as bone marrow, liver, heart, synovia, skin and others, apparently in a quiescent state [7]. It remains to be determined in what form the virus persists, whether as cryptic virions or as episomal or possibly integrating into the host genome, and whether the virus is functionally impaired, latent with the possibility of reactivation or persistently replicating at low levels [39]. In vitro, the virus can also infect a few semipermissive myeloblastoid cell lines, such as UT7/EpoS1 cells, giving rise to a restrictive pattern of replication [15], [16], while in cell lines such as U937 an abortive pattern of infection with limited genome expression has been reported [30]. Such two systems can be proposed as models for establishment of persistence, where a role for epigenetic regulation can be hypothesised and studied.

In our experiments, we investigated the presence of CpG dinucleotide methylation by performing analysis of bisulfite-modified DNA. Sequencing of bisulfite-modified, PCR amplified viral DNA can be employed for the direct detection of methylated cytosines within the full sequence of the region of interest, however this approach has the disadvantage that base representation in the amplification product is not linear and low-level methylation might therefore remain undetected. To overcome these limitations, a quantitative methylation specific real-time PCR assay was developed, achieving a quantitative determination of the methylation status of specific positions in the viral genome. While not being a genome-wide technique [40], our approach had the advantage of a targeted and accurate quantitative determination and ability to detect minimal frequencies of methylated DNA in relevant selected positions.

The effect of CpG methylation on viral DNA was initially assessed on transfected full-length, functionally competent cloned viral DNA [12], [26]. When measuring the degree of methylation of CpG dinucleotides within the left terminal region of the viral genome, a contrasting effect was observed, so that absence of substantial methylation of transfected unmethylated viral DNA was opposed to a limited but definite extent of demethylation of transfected methylated viral DNA. This last phenomenon can be a possible result of the balance between the replicative activity attributed to transfected viral DNA [26] and the accessibility of viral DNA to maintenance methylating enzymes, in a process comparable to demethylating replication. The lower specific transcriptional activity of transfected methylated viral DNA can be compatible with a possible silencing effect of CpG methylation on viral DNA. In fact, methylation of CpG residues within the left ITR and viral promoter can have a direct negative effect on the promoter activity, as determined by evaluation of expression of a reporter gene under control of viral sequences. Silencing of the viral promoter might be due to impaired interaction with cellular transcription factors [41], however, in the context of a complete viral genome other mechanisms might be involved, for example impaired interaction with viral NS protein in its transactivator function, or histone complexing exerting regulatory effects.

In addition to a measurable effect of CpG methylation on the viral expression profile following transfection experiments, we could also document the occurrence of CpG methylation and its possible effects in the course of in vitro infections of model cellular systems. In particular, we investigated the effects of cytosine methylation in CpG dinucleotides within the left terminal region of the viral genome in the course of in vitro infection experiments, focused on restrictive or nonpermissive environments such as UT7/EpoS1 and U937 cells, as model systems for the analysis of a possible epigenetic regulation leading to establishment of persistence. In the restrictive UT7/EpoS1 cells, the progressive loss of transcriptional and replicative activity of the viral genome was only at late times correlated to the establishment of low levels of CpG methylation. In the nonpermissive U937 cells, CpG methylation was more prominent. In this instance, cells could be infected giving rise to a non productive replicative cycle, and the viral genome persisted within cells at low, relatively stable levels with progressive accumulation of a high proportion of methylated DNA. We could not detect a measurable expression of the viral genome, as described by other investigators mainly occurring as a consequence of antibody-dependent enhancement of infection [30]. On the contrary, it appeared that absence of viral expression and genome replication were both correlated to increasing levels of CpG methylation of viral DNA.

Finally, cytosine methylation in CpG dinucleotides within the selected target region was also observed in viral DNA present in bioptic samples obtained from different tissues. This directly indicates the occurrence of CpG methylation of viral DNA in infected cells, that can possibly be indicative of the progressive establishment of persistence of the viral genome in tissues, and suggests a potential role for epigenetic mechanisms in regulating the course of natural infections. Further analysis conducted on larger groups of clinical samples will be required to investigate this eventuality.

From our experiments, it is possible to hypothesise that viral DNA, in its active state in the course of productive infections in permissive cellular environments, is not methylated to a significant extent, but that the absence of transcriptional and replicative activity of the viral genome as observed in nonpermissive cellular environments can be correlated to the progressive methylation of viral DNA, that in turn may contribute to silencing of the viral genome and establishment of viral persistence. Viral DNA can likely be complexed to histons, and be maintained as episome or, a possibility not experimentally demonstrated so far, may be integrated in the host genome, as it happens for AAV. These mechanisms would add possible further levels of epigenetic regulation to the viral expression profile. The progressive loss of viral DNA from infected cell cultures indicates its maintenance in episomal form in our experimental setting, whereas the possible integration of the viral genome into the host cell genome could not be directly investigated in our bioptic samples.

It should be also recalled that the occurrence of CpG methylation on viral DNA is also relevant for recognition of infecting agents operated by the innate immune systems, as unmethylated viral DNA can be a PAMP recognised by TLR9. A consensus sequence present in B19 genome interacts with TLR9 and has been shown to inhibit erythroid progenitor cells [42]. Methylation of viral DNA may also contribute to impairment of pathogenetic potential of the virus and induction of tolerance by the innate immune systems.

In conclusion, our experiments indicate the presence of an epigenetic level of regulation of viral genome expression, possibly linked to the silencing of the viral genome and contributing to the maintenance of the virus in tissues, a feature typical of parvoviruses. Further investigation will be needed to confirm and elucidate in detail the complex network of epigenetic regulation, involving not only CpG DNA methylation, but also histone complexing and modifications as well as nucleosome positioning, in different cellular environments. The picture of parvovirus B19 as a virus typically capable of lytic, acute infections has given place to a more complex landscape of a virus capable of diverse lytic, persistent or even latent/reactivating infections. Epigenetic regulation, as a viral adaptation to host cells, can be relevant to the balance and outcome of these different types of infection.
